# TRP36-ELISA for *E. canis* detection: Concordance with TaqMan real-time PCR and point-of-care testing

**DOI:** 10.1016/j.heliyon.2024.e39652

**Published:** 2024-10-22

**Authors:** Kitjawan Khumtub, Peeravit Sumpavong, Khomsan Satchasataporn, Chanon Fa-Ngoen, Sarawan Kaewmongkol, Gunn Kaewmongkol

**Affiliations:** a Department of Veterinary Technology, Faculty of Veterinary Technology, Kasetsart University, Bangkok, Thailand; b Queen Saovabha Memorial Institute, Thai Red Cross Society, Bangkok, Thailand; c Department of Companion Animals Clinical Sciences, Faculty of Veterinary Medicine, Kasetsart University, Bangkok, Thailand

**Keywords:** *Ehrlichia canis*, Recombinant TRP36, Enzyme-linked immunosorbent assay (ELISA), TaqMan real-time PCR, Point of care (POC) test, IgM, IgG, Early detection

## Abstract

*Ehrlichia canis*, a widely distributed tick-borne pathogen, requires prompt and accurate diagnosis for effective management. Real-time PCR serves as the reference standard for diagnosing *E. canis* infection, whereas serological tests are crucial for detecting antibody responses indicative of infection or post-exposure status. Early detection during the acute phase of the disease is essential for optimal treatment and recovery. *E. canis* Tandem Repeat Protein 36 (TRP36) elicits early acute phase responses. We developed an enzyme-linked immunosorbent assay (ELISA) using recombinant TRP36 to detect IgM and IgG antibodies against *E. canis*. We assessed the diagnostic agreement, sensitivity, and specificity of the rTRP36-ELISA and a commercial test kit (SNAP 4Dx Plus Test) with TaqMan Real-time PCR (qPCR) using the NCSS 2023 program version 23.0.1. Leftover serum samples from 32 dogs at the Veterinary Teaching Hospital were randomly selected and examined for *E. canis* infection using qPCR, rTRP36-ELISA, and SNAP 4Dx Plus Test. qPCR detected positive results in 12 (37.5 %), whereas ELISA and SNAP 4Dx Plus detected positive results in 10 (31.25 %) and 13 (40.62 %), respectively. Moderate agreement (κ = 0.545) was observed between rTRP36-ELISA and qPCR, and fair agreement (κ = 0.379) between SNAP 4Dx Plus and qPCR. Substantial agreement (κ = 0.79) was found between rTRP36-ELISA and SNAP 4Dx Plus. Compared to qPCR, the sensitivity and specificity of rTRP36-ELISA were 70 % and 77.27 %, respectively, compared to 53.85 % and 73.86 %, respectively, for SNAP 4Dx Plus. Our findings suggest that TRP36 is a promising antigen for *E. canis* serodiagnosis, potentially improving sensitivity and specificity.

## 1 Introduction

Ehrlichiosis is a canine vector-borne disease (CVBDs) transmitted by ticks that affects both humans and animals [1]. Canine monocytic ehrlichiosis (CME) is caused by *Ehrlichia canis,* an obligate intracellular gram-negative bacterium of the Alphaproteobacteria group, *Anaplasmataceae* family, and *Rickettsiales* order that commonly infects monocytes or macrophages. CME manifests in several stages, including acute, subclinical, and chronic phases [[2], [3], [4]]. The TaqMan real-time PCR assay offers several benefits, including the ability to diagnose diseases at an early stage, even before antibody production, and high sensitivity [5,6]. However, specialized equipment and highly experienced technicians are required for TaqMan real-time PCR assays, which are the disadvantages of this technique [7].

Serological diagnostic tests can determine both the current infection and the history of *E. canis* exposure by detecting specific immunoglobulin G (IgG) antibody responses against *E. canis*. To address serological cross-reactivity, recombinant proteins are used as specific antigens for enzyme-linked immunosorbent assays (ELISA), improving the specificity compared to indirect immunofluorescent assays (IFA) [8]. *E. canis* Tandem Repeat Protein 36 (TRP36) is a highly variable protein that plays a role in the immune response and contributes to the genetic diversity of *E. cani*s strains in various geographical areas. This glycoprotein exhibits diversity in numbers and sequences in the C-, N- terminals and tandem repeat regions. The TRP36 protein is highly conserved and specific within each *E. canis* strain and genogroup [9,10]. Phylogenetic analysis of the TRP36 protein in *E. canis* revealed the two distinct genetic groups in Thailand, corresponding to the US and Taiwanese genogroups [11,12].

Ehrlichial DNA was detected using real-time polymerase chain reaction (PCR) between 7 and 10 day after inoculation [13] and the findings by Beall et al. (2022) showed that experimentally infected dogs exhibited positive PCR results between 10 and 17 day after tick infestations [14]. Infected dogs displayed clinical signs 9–12 day after experimental infection [13]. The production of specific IgG antibodies against *E. canis* typically begins until 12–14 day after infection [15,16]. Moreover, IgG can persist in the bloodstream and maintain seropositivity after treatment or self-eradication of the infection [17,18]. The SNAP 4Dx assay (IDEXX Laboratories Inc., USA) utilizes two specific *E. canis* recombinant proteins, p30 and p30-1 [19]. This assay can detect antibodies as early as day 17 after inoculation [20]. As a first-generation screening tool, the SNAP 4Dx Point-of-Care (POC) ELISA was initially developed to identify dogs exposed to *E. canis* by detecting antibodies against the p30 and p30-1 proteins. In *E. canis* infections, the immune response specific to these p30 and p30-1 proteins may take several weeks post-infection to produce a detectable level of immunodominant antibodies. As a result, a dog might exhibit clinical signs and molecular evidence of infection (e.g., through PCR) before a serologic response can be detected by the POC test [14,20]. In an experiment with tick-transmitted *E. canis* infestations in six dogs, the second-generation test (SNAP 4Dx Plus Test, IDEXX Laboratories, Westbrook, ME, USA) detected seroconversion 3–20 day earlier to the first-generation test in four dogs [14]. The second-generation SNAP 4Dx Plus Test was developed for earlier detection and was compared with qPCR. However, in the early stage of the disease, only 3 out of 6 dogs tested using the SNAP 4Dx Plus showed agreement with qPCR [14]. The difference between first-generation and second-generation tests concerning earlier *Ehrlichia* antibody detection can be challenging to quantify in dogs naturally infected with *E. canis*, given the chronicity of *Ehrlichia* infections and the persistence of antibodies [21]. Prolonged IgG levels after treatment or during the chronic stage can further complicate diagnosis [22].TRP36 has been shown to increase the sensitivity and specificity of recombinant protein ELISAs compared to IFA during the early immune response, which detected the early phase of acute infection at day 14 and was highly specific to *E. canis* [23]. TRP36 serology can provide valuable serologic data on *E. canis* exposure and potential genotypes associated with infections [24,25].

A report in Thailand demonstrated that dog serum exhibited specific reactivity to the Thai recombinant TRP36 protein in the presence of IgM and IgG antibodies, using Western blot analysis [26]. Furthermore, western blotting using rTRP36 tested in dog serum showed competitive performance compared to the SNAP 4Dx test (first-generation test) [26]. Analysis of specific IgM and IgG levels could provide insights into the continuous re-exposure to *E. canis* in infected dogs living in endemic areas. This study aimed to develop a novel ELISA based on recombinant TRP36 protein to detect specific IgM and IgG antibodies against *E. canis* obtained from local Thai strains, and to assess the diagnostic agreement, sensitivity, and specificity of the rTRP36-ELISA and a commercial test kit (SNAP 4Dx Plus Test) with TaqMan Real-time PCR (qPCR).

## 2 Materials and methods

### 2.1 Ethics statement

The Animals for Scientific Purposes Act (A.D. 2015) of Thailand was adhered to and permission was obtained from the Laboratory Animal Committee of Kasetsart University, Faculty of Veterinary Medicine (approval ID: ACKU63-VET-038). The authors of this study obtained authorization (approval number U1-00325-2558) to use animals for scientific studies.

### 2.2 Samples

Thirty-two whole blood and serum samples from dogs were randomly obtained from routine leftover samples at the hematology laboratory of Kasetsart Veterinary Teaching Hospital, Faculty of Veterinary Medicine, Kasetsart University as described in a previous study [27]. Blood and serum samples were stored at −20 °C until analysis. The blood and serum samples used in our study were randomly selected from dogs presenting with non-specific clinical signs. These samples were leftover from routine hematological work at the veterinary teaching hospital, and therefore, the dogs' health status was not specifically classified as either healthy or infected at the time of collection. The data collected included age, breed, sex, and hematological parameters (Table S1).

### 2.3 TaqMan real-time polymerase chain reaction (qPCR) for *E. canis* detection

A total of 32 dog blood samples were analyzed using the TaqMan Real-time PCR technique, as previously described [5]. The TaqMan Real-time PCR (qPCR) targets the *dsb* gene. The qPCR reaction mixture (20 μL) contained 10 μL of × 5 HOT FIREPol Probe qPCR Mix Plus (Solis BioDyne, Tartu, Estonia), 1 μM of each primer, DSB-321 5′-TTGCAAAATGA-TGTCTGAAGATATGAAACA-3′ and DSB-671 5′-GCTGCTCCACCAATAAATGTAT-CYCCTA-3′, and the TaqMan probes; FAM-5′-AGCTAGTGCTGCTTGGGCAACTTTGAG-TGAA-BHQ1-3’. The qPCR was performed by a Real-time PCR CFX96 machine (Bio-Rad Laboratories, Hercules, CA, USA). The PCR cycling conditions consisted of 95 °C for.

15 min, followed by 45 cycles of 95 °C for 15 s and 60 °C for 1 min [5].

### 2.4 Evaluation of *E. canis* infection using the SNAP 4Dx Plus Test

The 32 dog serum samples were evaluated using the SNAP 4Dx Plus Test (IDEXX Laboratories Inc., USA) following the instructions provided in the product information.

### 2.5 Recombinant TRP36-ELISA

Recombinant TRP36 protein (originated from amino acid sequence accession number ALU10976.1) was cloned and expressed using pBAD/TOPO® ThioFusion™ and transformed into *Escherichia coli* TOP10 (InvitrogenTM, USA) according to the manufacturer's instructions (Synbio Technologies, USA) [26]. The cells were harvested by centrifugation at 10,000 rpm for 10 min and resuspended in a solution containing 50 mM sodium phosphate, pH 8.0, 300 mM sodium chloride, and 6 M urea. The suspension was lysed by sonication, and the soluble protein was separated by centrifugation at 10,000 rpm for 10 min at 4 °C. The protein solution was subjected to immobilized metal-ion affinity chromatography (GE Healthcare, USA). The protein was eluted with a stepwise gradient of 50–500 mM imidazole in a solution containing 50 mM sodium phosphate, pH 8.0, and 300 mM sodium chloride. The protein fractions were analyzed by 12 % SDSPAGE [26]. The rTRP36 was suspended in molecular biology-grade water (0.1 mg/mL). Flat-bottom 96 well microtiter plates (Greiner Bio-One, Germany) were coated with recombinant TRP36 protein (1.0 μg/well; 50 μl) dissolved in sterile phosphate-buffered saline buffer pH 7.4 (Takara Bio, Japan) and incubated at 4 °C overnight. The next day, ELISA wells with antigen were washed four times with 300 μl phosphate-buffered saline with Tween 20 (0.1 %) (PBST; pH 7.4). Plates were blocked with PBST with 5 % skim milk; (Becton, Dickinson and Company, France) at 100 μl per well and washed four times. The serum 50 μl (diluted 1:100) in PBST with 5 % skim milk was added to each well and incubated for 1 h at 37 °C. Plates were washed four times and 50 μl of the secondary goat anti-dog IgG/IgM horseradish peroxidase (HRP) conjugate antibody (KPL, USA) diluted in PBST with 5 % skim milk (1:1000) was added. Plates were incubated for 1 h at 37 °C, washed, and KPL SureBlue™ TMB Microwell Peroxidase Substrare (Seracare Life Sciences, USA) (100 μl) was added to each well. The plates were then incubated in a dark chamber at room temperature for 15 min before measuring the optical density at a wavelength of 620 nm using an ELISA plate reader (Bio-Rad Laboratories, USA). We determined a cutoff value for IgM and IgG detection as three standard deviations from the mean optical density of the non-reactive serum (serum that displayed negative qPCR and SNAP 4Dx Plus Test results). Samples with an O.D. reading of 0.32 unit was considered for positive IgM detection. A reading of 0.72 OD units was considered positive for IgG detection. If either the IgM or IgG test yielded positive results, the overall result was considered positive.

### 2.6 Statistical analysis

The NCSS 2023 program version 23.0.1 was used to perform statistical analysis of the data obtained from the TaqMan Real-time PCR, rTRP36-ELISA, and SNAP 4Dx Plus Test results. Agreement among all tests (TaqMan Real-time PCR, rTRP36-ELISA, and SNAP 4Dx Plus Test) was analyzed using Cohen's kappa (κ) test. Interpretation of the κ value was as follows: κ values = 0 indicates no agreement between two tests or raters that would be expected by random chance. The agreement is considered poor; κ = 0.01–0.20 indicates a very low level of agreement that would be expected by chance alone. The agreement is considered none to slight agreement; κ = 0.21–0.40 indicates some agreement but not much beyond chance. The agreement is considered fair. However, the agreement is still relatively weak; κ = 0.41–0.60 indicates the tests or raters agree more than they would by chance, but there is still a significant amount of disagreement. The agreement is considered moderate agreement; κ = 0.61–0.80 indicates a stronger level of agreement, which is substantial and indicates that the tests or raters generally agree well; and κ = 0.81–1.00 indicates almost perfect agreement, where the two tests or raters agree almost completely [28]. The Z-scores were calculated to validate the strength and statistical significance of these Cohen's kappa agreements (supplementary files). Sensitivity, specificity, positive predictive value (PPV), negative predictive value (NPV), positive likelihood ratio, and negative likelihood ratio were analyzed between a reference standard tool, TaqMan Real-time PCR and serological tests including rTRP36-ELISA, and SNAP 4Dx Plus Test.

## 3 Results

### 3.1 Evaluation of *E. canis* infection using TaqMan real-time PCR, recombinant TRP36-ELISA, and SNAP 4Dx Plus Test

A total of 32 samples were analyzed. TaqMan Real-time PCR results showed that 12 of 32 (37.5 %) samples tested positive. Among these 12 qPCR-positive samples, concordance among the three tests was found in seven samples. The remaining five samples (15.625 %) were positive only by qPCR (Table 1). The SNAP 4Dx Plus Test (IDEXX Laboratories, Westbrook, ME, USA) showed that 13 of the 32 (40.62 %) samples tested positive. Ten of these samples (31.25 %) were also tested positive by rTRP36-ELISA. The rTRP36-ELISA results indicated that 10 of the 32 (31.25 %) samples tested positive. Among these positive samples, six (18.75 %) were positive for IgM and nine (28.12 %) were positive for IgG. One sample (3.12 %) tested positive for IgM only, four (12.5 %) tested positive for IgG only, and five (15.6 %) tested positive for both IgM and IgG (Table 1).

**Table 1:** Result from the TaqMan Real-time PCR, rTRP36-ELISA, and SNAP 4Dx Plus Test Kits on the serum and blood samples of 32 dogs.

Sample No.	Lab No.	TaqMan Real-time PCR	rTRP36-ELISA	SNAP 4Dx Plus Test
1	1	+	IgM + IgG	+
12	2	–	–	–
115	3	–	–	–
117	4	–	IgM	+
121	5	+	IgG	+
129	6	+	–	–
133	7	+	–	–
138	8	+	IgG	+
153	9	–	–	–
158	10	+	–	–
167	11	+	IgM + IgG	+
186	12	+	IgG	+
194	13	–	–	–
197	14	+	–	–
211	15	–	–	+
213	16	–	–	+
214	17	–	–	–
222	18	–	–	–
227	19	+	IgM + IgG	+
349	20	+	IgG	+
321	21	–	IgM + IgG	+
177	22	–	–	+
280	23	+	–	–
362	24	–	–	–
369	25	–	–	–
380	26	–	IgM + IgG	+
386	27	–	–	–
293	28	–	–	–
363	29	–	–	–
381	30	–	–	–
406	31	–	–	–
431	32	–	–	–
**Total**	**32**	**12**	**10**	**13**

### 3.2 Statistical analysis

The data obtained from TaqMan Real-time PCR, rTRP36-ELISA, and SNAP 4Dx Plus Test results were analyzed statistically using the NCSS 2023 program version 23.0.1. The agreement among all tests was evaluated using Cohen's kappa (κ) test.

The results of each serological test, including rTRP36-ELISA and SNAP 4Dx Plus, were compared with the TaqMan Real-time PCR results (Table 2). The rTRP36-ELISA exhibited moderate agreement with qPCR, with κ = 0.545 (95 % CI; 0.460–0.623). The SNAP 4Dx Plus Test demonstrated fair agreement with qPCR, with κ = 0.379 (95 % CI; 0.296–0.462). When comparing rTRP36-ELISA with SNAP 4Dx Plus Test, substantial agreement existed between the two tests, with κ = 0.793 (95 % CI; 0.738–0.848) (Table 3).

**Table 2:** Results of positive and negative samples by rTRP36-ELISA and SNAP 4Dx Plus Test compared with TaqMan Real-time PCR.

Test results	TaqMan Real-time PCR results		Kappa value (95%CI)	Degree of agreement
	positive	negative		
**rTRP36-ELISA**			0.545 (0.460–0.623)	moderate
positive	7	3		
negative	5	17		
Total (n = 32)				
**SNAP 4Dx Plus Test**			0.379 (0.296–0.462)	fair
positive	7	6		
negative	5	14		
Total (n = 32)				

CI = confidence interval.

**Table 3:** Results of positive and negative samples by SNAP 4Dx Plus Test compared with rTRP36-ELISA.

Test results	rTRP36-ELISA results		kappa value (95%CI)	degree of agreement
	positive	Negative		
**SNAP 4Dx Plus Test**			0.793 (0.738–0.848)	substantial
positive	10	3		
negative	0	19		
Total (n = 32)				

CI = confidence interval.

### 3.3 Diagnostic sensitivity and specificity

A comparison of the serological tests with TaqMan Real-time PCR is shown in Table 4. The sensitivities of rTRP36-ELISA and SNAP 4Dx Plus Test were 70.00 % (95 % CI; 34.45–93.33 %) and 53.85 % (95 % CI; 25.13–80.78 %), respectively. The specificities of rTRP36-ELISA and SNAP 4Dx Plus Test were 77.27 % (95 % CI; 54.63–92.18 %) and 73.68 % (95 % CI; 48.80–90.85 %), respectively. Both tests had an equal positive predictive value (58.33 %, 95 % CI; 27.67–84.83 %). The rTRP36-ELISA had a negative predictive value of 85.00 %, compared to that of 70.00 % for the SNAP 4Dx Plus Test. The positive and negative likelihood ratios of the rTRP36-ELISA were 3.08 and 0.38, respectively, whereas those for the SNAP 4Dx Plus Test were 2.04 and 0.62, respectively.

**Table 4:** Diagnostic assessment of rTRP36-ELISA and SNAP 4Dx Plus Test for canine monocytic ehrlichiosis compared with TaqMan Real-time PCR.

Diagnostic assessment	rTRP36-ELISA (95%CI)	SNAP 4Dx Plus Test (95%CI)
Sensitivity (%)	70.00 (34.75–93.33)	53.85 (25.13–80.78)
Specificity (%)	77.27 (54.63–92.18)	73.68 (48.80–90.85)
Positive predictive value (%)	58.33 (27.67–84.83)	58.33 (27.67–84.83)
Negative predictive value (%)	85.00 (62.11–96.79)	70.00 (45.72–88.11)
Positive likelihood ratio	3.08 (1.28–7.35)	2.04 (0.82–5.05)
Negative likelihood ratio	0.38 (0.14–1.02)	0.62 (0.32–1.19)

## 4 Discussion

This study aimed to evaluate the diagnostic performance of rTRP36-ELISA and SNAP 4Dx Plus assay in comparison with TaqMan Real-time PCR for detecting *Ehrlichia canis* infection in dogs. Our findings highlight the potential of the rTRP36-ELISA as a reliable serological tool for the early and accurate detection of *E. canis*. Dog serum samples were randomly selected and tested using the novel rTRP36-ELISA kit and a commercially available test kit (SNAP 4Dx Plus). Blood samples obtained from the same dogs were examined for *E. canis* infection using TaqMan real-time PCR. The results of the novel rTRP36-ELISA indicated that naturally *E. canis*-infected dogs produced IgM antibodies against TRP36. These findings support the successful detection of IgM antibodies in dogs infected with *E. canis* by Western blot analysis, as shown in a previous study [26]. This observation aligns with humoral immune response theories, which state that IgM is the primary immunoglobulin produced during B cell formation. IgM antibodies play a crucial role in initial immunological reactions and are often used to diagnose acute exposure to immunogens or infections [29]. Historically, IgM antibodies have been regarded as reliable markers for acute infections across various pathogens, such as Dengue and Lyme borreliosis [30,31]. In several infectious diseases, the emergence of IgG antibodies often follows the decline of IgM antibodies and persists in the bloodstream [32,33]. The potential of using specific IgM for the early diagnosis of CME is closer to its practical application than that in previous reports.

The current infection status of the dogs in this study was assessed based on positive results for qPCR, for IgM or IgG antibodies, and for the SNAP 4Dx Plus Test. The possibility arises that dogs tested positive for both IgM and IgG might have been exposed to *E. canis* multiple times. Various studies have reported that Thailand is an endemic area for CME, with *E. canis* detected in several regions of the country [[34], [35], [36]]. According to a study, *E. canis* was commonly detected in both healthy and sick dogs as well as in brown dog ticks [36]. Multiple exposures to *E. canis* can occur in dogs living in this region. This continuous exposure can lead to repeated or persistent infections, complicating diagnosis and treatment.

Dogs that test positive only for IgM may be experiencing a primary infection, indicating new or initial exposure. According to a previous report on dengue infections, the presence of IgM alone can be interpreted as a recent primary infection [30]. Additionally, positive IgM results with negative molecular results may be due to a low bacterial load that has yet to reach the threshold detectable by molecular analyses. A study of dengue infection indicated that the dynamics of viral multiplication and antibody generation occur at different times. As a result, the number of pathogens fluctuates to a lower viral load and antibodies begin to form in the body [32]. Sumpavong et al. (2022) established a threshold for quantitative polymerase chain reaction (qPCR), revealing that the minimum concentration of target templates required for positive results was a dilution of 10^−7^, indicating a significant limit of molecular detection [5].

SNAP 4Dx Plus tested positive in three dogs (9.38 %), with negative results from qPCR and rTRP36-ELISA. We hypothesized that these dogs would exhibit persistent and prolonged IgG antibody levels. Antibody titers against *E. canis* can persist for several months or years. Despite this, the dog may have been treated and recovered [16], demonstrating a notable limitation of serological detection. Meanwhile, dogs in this study that tested positive only by qPCR may have been in the infective stage of the disease, yet their antibody levels were insufficient to indicate seropositivity. Low antibody levels have been reported during the early acute phase of ehrlichiosis in dogs, which might be one of the possible reasons for negative seropositivity [23,37]. Therefore, our further study focuses on CME's recovery phase (after the treatment stage). Our hypothesis is that the antibody titers detected by TRP36-ELISA are more rapidly declining than SNAP 4Dx Plus at the recovery stage after treatment.

The rTRP36-ELISA showed a moderate level of concordance with qPCR (κ = 0.545), whereas the SNAP 4Dx Plus Test demonstrated a fair level of agreement (κ = 0.379). The results of this study indicated a slight discordance between rTRP36 ELISA and SNAP 4Dx Plus in diagnosing infectious diseases compared with qPCR. However, a significant level of agreement (κ = 0.79) was observed between the rTRP36-ELISA and SNAP 4Dx Plus, indicating comparable efficacy in detecting specific antibodies to *E. canis*.

The rTRP36-ELISA exhibited higher diagnostic measurement values, with a sensitivity of 70.00 %, than SNAP 4Dx Plus (sensitivity of 53.85 %). Consequently, the ELISA assay provided a greater number of true positive results than the SNAP 4Dx Plus. However, both assays demonstrated similar levels of false-negative results, as evidenced by their specificity values of 77.27 % and 73.68 %. Both tests revealed equal positive predictive values (58.33 %), indicating similar ability to accurately identify positive results. Simultaneously, rTRP36-ELISA exhibited a greater capacity to accurately identify negative results, as evidenced by its larger negative predictive value (85.00 % vs. 70.00 %). The positive likelihood ratio (PLR) indicated that the rTRP36-ELISA performed better in detecting *E. canis* than SNAP 4Dx Plus (3.08 vs. 2.04). Conversely, the negative likelihood ratio (NLR) demonstrated that the rTRP36-ELISA outperformed SNAP 4Dx Plus in determining the absence of true infection in dogs (0.38 vs. 0.62).

However, the sample size of this study was small. Future research should be conducted with larger populations to develop point-of-care tests. Cross-reactivity may have occurred in this study with other closely related species, such as *Ehrlichia* spp., *Anaplasma* spp., or other tick-borne pathogens. Further studies in Thailand are required to test cross-reactivity with other closely related pathogens. In addition, the dynamics of IgM in *Ehrlichia* species require clarification. McBride et al. (2003) used enzyme-linked immunosorbent assays (ELISA) to assess the presence of IgM antibodies in *E. canis* whole-cell lysates. Low levels of IgM were detected in the four experimental groups of dogs infected with *E. canis* on day 28 of the acute phase of canine ehrlichiosis. Additionally, dogs were confirmed to have negative IgM antibody levels on days 7 and 14 [38]. According to Neitz et al. (1986), IgM antibodies were present on day 4 following infection, however, became undetectable by day 7 in cases of *E. ruminantium* infection [39]. IgM response might have had a short duration, reaching its peak within seven days between sample collections and then decreasing to undetectable levels. Confirmation of an *E. canis* diagnosis requires the application of different procedures, and the interpretation of IgM information should be approached with caution.

The development of the recombinant TRP36-ELISA in this study represents a novel approach for detecting *E. canis* antibodies. rTRP36-ELISA has great potential as an effective tool for detecting infectious diseases during the initial acute phase of *E. canis* infection owing to its rapid antibody response, which may not persist for an extended period. By accurately differentiating between ongoing and resolved infections, veterinarians can more effectively monitor the success of treatments and the resolution of the disease, especially in managing chronic and recurrent infections. Further investigation is needed to explore the efficacy of the TRP36 protein, potentially paving the way for its use as a viable point-of-care (POC) test within the broader context of the Thai infected dog community in the near future.

## 5 Conclusion

Our study underscores the potential of the TRP36 protein as a promising alternative antigen for the serodiagnosis of *Ehrlichia canis* infection, potentially enhancing the sensitivity and specificity of diagnostic approaches. This is particularly relevant in Southeast Asia, where *E. canis* is endemic and dogs face year-round exposure to the pathogen. Developing an efficient and cost-effective device capable of rapidly identifying specific *E. canis* strains in this tropical region is crucial. Utilizing TRP36 serology to detect specific IgM and IgG antibodies offers advantages for identifying the early phase of acute infection and distinguishing between new infections, previous exposure, and re-exposure to *E. canis*. Future advancements in serological point-of-care (POC) tests for *E. canis* detection could benefit from innovative ELISAs targeting both IgM and IgG using the TRP36 protein, thus improving canine health management and public health strategies against tick-borne diseases.

## CRediT authorship contribution statement

**Kitjawan Khumtub:** Writing – original draft, Investigation. **Peeravit Sumpavong:** Resources, Investigation. **Khomsan Satchasataporn:** Resources. **Chanon Fa-Ngoen:** Validation, Resources. **Sarawan Kaewmongkol:** Supervision, Methodology, Formal analysis. **Gunn Kaewmongkol:** Writing – review & editing, Supervision, Conceptualization.

## Data and code availability statement

Data included in article/supplementary material is referenced in the article.

## Declaration of generative AI and AI-assisted technologies in the writing process

Grammarly software was used for English grammar editing in some sentences.

## Declaration of competing interest

The authors declare that they have no known competing financial interests or personal relationships that could have appeared to influence the work reported in this paper.

## References

[1] Bouza-Mora L. (2017). Novel genotype of *Ehrlichia canis* detected in samples of human blood bank donors in Costa Rica. Ticks and Tick-borne Diseases.

[2] Buhles W.C., Huxsoll D.L., Ristic M. (1974). Tropical canine Pancytopenia: clinical, hematologic, and serologic response of dogs to *Ehrlichia canis* infection, tetracycline Therapy, and Challenge inoculation. J. Infect. Dis..

[3] Harrus S. (1997). Canine monocytic ehrlichiosis: a retrospective study of 100 cases, and an epidemiological investigation of prognostic indicators for the disease. VetRecord.

[4] Rodríguez-Alarcón C.A. (2020). Demonstrating the presence of *Ehrlichia canis* DNA from different tissues of dogs with suspected subclinical ehrlichiosis. Parasites Vectors.

[5] Sumpavong P. (2022). Systematic evaluation of TaqMan real-time polymerase chain reaction assays targeting the dsb and gltA loci of *Ehrlichia canis* in recombinant plasmids and naturally infected dogs. Vet. World.

[6] Nkosi N.F., Oosthuizen M.C., Quan M. (2022). Development and validation of a TaqMan® probe- based real-time PCR assay for detection of *Ehrlichia canis*. Ticks and Tick-borne Diseases.

[7] Kaltenboeck B., Wang C. (2005). Advances in real-time PCR: application to clinical laboratory diagnostics. Adv. Clin. Chem..

[8] McBride J.W. (2001). Immunodiagnosis of *Ehrlichia canis* infection with recombinant proteins. J. Clin. Microbiol..

[9] Zhang X. (2008). Genetic and antigenic diversities of major immunoreactive proteins in globally distributed *Ehrlichia canis* strains. Clin. Vaccine Immunol..

[10] Hsieh Y.-C. (2010). Detection and characterization of four novel genotypes of *Ehrlichia canis* from dogs. Vet. Microbiol..

[11] Kaewmongkol G. (2017). Association of *Ehrlichia canis*, Hemotropic *Mycoplasma* spp. and *Anaplasma platys* and severe anemia in dogs in Thailand. Vet. Microbiol..

[12] Nambooppha B. (2018). Two different genogroups of *Ehrlichia canis* from dogs in Thailand using immunodominant protein genes. Infect. Genet. Evol..

[13] Baneth G. (2009). Longitudinal quantification of *Ehrlichia canis* in experimental infection with comparison to natural infection. Vet. Microbiol..

[14] Beall M.J. (2022). An improved point-of-care ELISA for the diagnosis of Anaplasmosis and ehrlichiosis during the acute phase of tick-borne infections in dogs. Top. Companion Anim. Med..

[15] Harrus S. (1998). Therapeutic Effect of doxycycline in experimental subclinical canine monocytic ehrlichiosis: evaluation of a 6-week course. J. Clin. Microbiol..

[16] Harrus S. (1998). Amplification of ehrlichial DNA from dogs 34 months after infection with *Ehrlichia canis*. J. Clin. Microbiol..

[17] Waner T. (2001). Significance of serological testing for ehrlichial diseases in dogs with special emphasis on the diagnosis of canine monocytic ehrlichiosis caused by *Ehrlichia canis*. Vet. Parasitol..

[18] Hegarty B.C. (2009). Clinical relevance of annual screening using a commercial enzyme-linked immunosorbent assay (SNAP 3Dx) for canine ehrlichiosis. J. Am. Anim. Hosp. Assoc..

[19] Ohashi N. (1998). Cloning and characterization of multigenes encoding the immunodominant 30-kilodalton major outer membrane proteins of *Ehrlichia canis* and application of the recombinant protein for serodiagnosis. J. Clin. Microbiol..

[20] Moroff S. (2014). Use of an automated system for detection of canine serum antibodies against *Ehrlichia canis* glycoprotein 36. J. Vet. Diagn. Invest..

[21] Richardson S.S. (2023). A second-generation, point-of-care immunoassay provided improved detection of *Anaplasma* and *Ehrlichia* antibodies in PCR-positive dogs naturally infected with *Anaplasma* or *Ehrlichia* species. J. Vet. Diagn. Invest..

[22] Gaunt S.D. (2010). Experimental infection and co-infection of dogs with *Anaplasma platys* and *Ehrlichia canis*: hematologic, serologic and molecular findings. Parasites Vectors.

[23] Cárdenas A.M. (2007). Enzyme-linked immunosorbent assay with conserved immunoreactive glycoproteins gp36 and gp19 has enhanced sensitivity and provides species-specific immunodiagnosis of *Ehrlichia canis* infection. Clin. Vaccine Immunol..

[24] Aguiar D.M. (2016). Detection of genotype-specific *Ehrlichia canis* exposure in Brazilian dogs by TRP36 peptide ELISA, Ticks and Tick-borne. Diseases.

[25] Arroyave E. (2020). *Ehrlichia canis* TRP36 diversity in naturally infected-dogs from an urban area of Colombia. Ticks and Tick-borne Diseases.

[26] Kaewmongkol S. (2020). Detection of specific IgM and IgG antibodies in acute canine monocytic ehrlichiosis that recognize recombinant gp36 antigens. Heliyon.

[27] Fa-Ngoen C. (2024). Serological detection of *Rickettsia* spp. and evaluation of blood parameters in pet dogs and cats from Bangkok and neighboring provinces. PLoS One.

[28] McHugh M.L. (2012). Interrater reliability: the kappa statistic. Biochem. Med..

[29] Schroeder H.W., Cavacini L. (2010). Structure and function of immunoglobulins. J. Allergy Clin. Immunol..

[30] Rubens Costa Lima J. (2012). Interpretation of the presence of IgM and IgG antibodies in a rapid test for dengue: analysis of dengue antibody prevalence in Fortaleza City in the 20th year of the epidemic. Rev. Soc. Bras. Med. Trop..

[31] Joyner G. (2022). Introduction of IgM testing for the diagnosis of acute Lyme borreliosis: a study of the benefits, limitations and costs. Eur. J. Clin. Microbiol. Infect. Dis..

[32] Guzman M.G. (2010). Dengue: a continuing global threat. Nat. Rev. Microbiol..

[33] Hou H. (2020). Detection of IgM and IgG antibodies in patients with coronavirus disease 2019. Clinical and Translational Immunology.

[34] Piratae S. (2015). Molecular detection of *Ehrlichia canis, Hepatozoon canis* and *Babesia canis vogeli* in stray dogs in Mahasarakham province, Thailand. Annals of Parasitology.

[35] Liu M. (2016). Molecular survey of canine vector-borne diseases in stray dogs in Thailand. Parasitol. Int..

[36] Eamudomkarn C. (2022). Prevalence of *Ehrlichia-, Babesia-*, and *hepatozoon-*infected brown dog ticks in Khon kaen province, Northeast Thailand. Vet. World.

[37] Ndip L.M. (2005). Ehrlichial infection in Cameroonian canines by *Ehrlichia canis* and *Ehrlichia ewingii*. Vet. Microbiol..

[38] McBride J.W. (2003). Kinetics of antibody response to *Ehrlichia canis* immunoreactive proteins. Infect. Immun..

[39] Neitz A.W. (1986). Detection *of Cowdria ruminantium* antigen and antibody during the course of heartwater disease in sheep by means of an enzyme-linked immunosorbent assay. Onderstepoort J. Vet. Res..

