# Supplementary material for: TRP36-ELISA for E. canis detection: Concordance with TaqMan real-time PCR and point-of-care testing
Source: Heliyon. 2024 Oct 22;10(21):e39652. doi: 10.1016/j.heliyon.2024.e39652 (PMC11550044; doi:10.1016/j.heliyon.2024.e39652)
Supplement: Multimedia component 3 [file mmc3.pdf]

This report is for evaluation purposes only.
There are 25 days remaining in your free trial (Expires on 1/19/2024).

Cross Tabulation Report

Dataset	C:\Users\Sarawan\Desktop\NCSS_ELISAgp36\NCSS Kappa.xlsx
Frequency	Case
Row Variable	rtPCR
Column Variable	SNAP

Kappa Estimation
(rtPCR by SNAP)

Statistic	Value	Asymptotic Std. Error	95% Lower Conf. Limit	95% Upper Conf. Limit
Kappa	0.379	0.042	0.296	0.462
Weighted Kappa (Linear)	0.379	0.042	0.296	0.462
Weighted Kappa (Quadratic)	0.379	0.042	0.296	0.462
Maximum-Adjusted Kappa*	0.464			

* Maximum Kappa with the Observed Marginal Totals = 0.816

Kappa Hypothesis Tests
(rtPCR by SNAP)

H0: Kappa = 0
H1: Kappa > 0 (One-Sided)
H1: Kappa ≠ 0 (Two-Sided)

Test	Value	Asymptotic Std. Error under H0	Z	One-Sided Prob Level	Two-Sided Prob Level
Kappa	0.379	0.043	8.852	0.0000	0.0000
Weighted Kappa (Linear)	0.379	0.043	8.852	0.0000	0.0000
Weighted Kappa (Quadratic)	0.379	0.043	8.852	0.0000	0.0000

This report is for evaluation purposes only.
There are 25 days remaining in your free trial (Expires on 1/19/2024).

Cross Tabulation Report

Dataset	C:\Users\Sarawan\Desktop\NCSS_ELISAgp36\NCSS Kappa.xlsx
Frequency	Case
Row Variable	rtPCR
Column Variable	SNAP

Procedure Input Settings

Autosaved Settings File
C:\Users\Sarawan\Documents\NCSS 2023\Procedure Settings\Autosave\Contingency Tables (Crosstabs - Chi-Square Test) - Autosaved 2023_12_26-17_10_5.t164

Variables Tab

Type of Data Input: Columns in the Database

-- Categorical Table Variables ---

Row Variable(s): rtPCR
Create Other Row Variables from Numeric Data: Unchecked
Column Variable(s): SNAP
Create Other Column Variables from Numeric Data: Unchecked

-- Frequency (Count) Variable ---

Frequency Variable: Case

Breaks Tab

-- Grouping (Break) Variables ---

Number of Grouping Variables: 0

Missing Values Tab

-- Missing Value Options ---

Missing Value Inclusion: Delete All
Label for Missing Values: Missing

·· Data Values to be Treated as "Missing" ···

Missing Value 1: <Empty>
Missing Value 2: <Empty>
Missing Value 3: <Empty>
Missing Value 4: <Empty>
Missing Value 5: <Empty>

Reports Tab

-- Data Summary ---

Data Summary Report: Unchecked

-- Contingency Tables ---

Show Individual Tables: Unchecked
Show Combined Table: Unchecked

This report is for evaluation purposes only.
There are 25 days remaining in your free trial (Expires on 1/19/2024).

Cross Tabulation Report

Dataset	C:\Users\Sarawan\Desktop\NCSS_ELISAgp36\NCSS Kappa.xlsx
Frequency	Case
Row Variable	rtPCR
Column Variable	SNAP

Procedure Input Settings (Continued)

Reports Tab (Continued)

-- Table Statistics and Tests --

Tests for Row-Column Independence (Pearson's Chi-Square, Fisher's Exact)	Unchecked
Tests for Trend in Proportions (Cochran-Armitage) [2×k Tables]	Unchecked
McNemar Test [k×k Tables]	Unchecked
Kappa and Weighted Kappa Tests for Inter-Rater Agreement [k×k Tables]	Checked
Confidence Level:	95
Association and Correlation Statistics	Unchecked

·· Alpha for Tests ··

Alpha:	0.05

Multiple Comparisons Tab

-- General Options --

Success Category:	Second Category
Alpha for MC Tests and C.I.'s:	0.05

-- Pairwise Multiple Comparisons [2×k Tables] --

·· Differences ··

Pairwise Wald Tests and C.I.'s of Differences	Unchecked
Pairwise Score Tests and C.I.'s of Differences	Unchecked
Pairwise Tests of Differences using the Angular Transformation	Unchecked

·· Odds Ratios ··

Pairwise Score Tests and C.I.'s of Odds Ratios	Unchecked

-- Multiple Comparisons Versus a Control Group [2×k Tables] ---

Control Group:	Last Group

·· Differences ··

Two-Sided Dunnett Tests and C.I.'s versus a Control	Unchecked
Two-Sided Bonferroni Tests and C.I.'s versus a Control	Unchecked
Lower One-Sided Dunnett Tests and C.I.'s versus a Control	Unchecked
Upper One-Sided Dunnett Tests and C.I.'s versus a Control	Unchecked

·· Odds Ratios ··

Dunnett Score Tests and C.I.'s of Odds Ratios	Unchecked

This report is for evaluation purposes only.
There are 25 days remaining in your free trial (Expires on 1/19/2024).

Cross Tabulation Report

Dataset	C:\Users\Sarawan\Desktop\NCSS_ELISAgp36\NCSS Kappa.xlsx
Frequency	Case
Row Variable	rtPCR
Column Variable	SNAP

Procedure Input Settings (Continued)

Report Options Tab

-- Table Formatting --

Column Justification:	Right
Column Widths:	Autosize to Equal Minimum Width
Wrap Column Headings onto Two Lines	Checked

-- Decimal Places --

Counts:	0
Percentages:	2
Omit Percent Sign after Percentages	Unchecked
Expected Counts:	1
Statistics (Kappa, SE, etc.):	3
Test Statistics (Chi-Square, Z):	3
P-Values:	4
Row / Column Labels:	Auto (Up to 7)
Proportions:	4
Odds Ratios:	4

Plots Tab

-- Bar Charts --

Show Bar Charts	Unchecked

-- Line Charts --

Show Line Charts	Unchecked

-- General Plot Options --

Show Break as Title	Checked
